# Bilateral Acromioclavicular Septic Arthritis as an Initial Presentation of *Streptococcus pneumoniae* Endocarditis

**DOI:** 10.1155/2014/313056

**Published:** 2014-06-01

**Authors:** Neda Hashemi-Sadraei, Rohan Gupta, Jorge D. Machicado, Rukma Govindu

**Affiliations:** Department of Internal Medicine, The University of Texas Health Science Center at Houston, 6431 Fannin Street, MSB 1.134, Houston, TX 77030, USA

## Abstract

Infective endocarditis (IE) is infrequently associated with septic arthritis. Moreover, septic arthritis of the acromioclavicular (AC) joint is rarely reported in the literature. We report a case of *Streptococcus pneumoniae* IE in a patient who presented with bilateral AC joint septic arthritis and we review the literature on the topic.

## 1. Introduction

Infective endocarditis (IE) is frequently associated with osteoarticular manifestations. Though, septic arthritis associated with IE is rare [1]. Moreover, septic arthritis of the acromioclavicular (AC) joint is rarely reported in the literature, with majority of the cases described in patients with underlying predisposing conditions. Herein, we report a case of a 43-year-old man with bilateral AC joint septic arthritis due to* Streptococcus pneumoniae* who was found to have infective endocarditis (IE). This is the first case reported of IE caused by* S. pneumoniae* presenting with bilateral septic arthritis of the AC joint, and we review the literature on the topic.

## 2. Case Report

A 43-year-old African American man presented to the emergency department with five days of arthralgias. The patient initially developed bilateral shoulder pain and swelling, followed by bilateral hip pain and swelling of the third digit of the right hand. He recalled local trauma to this finger while playing basketball three weeks earlier, causing transient swelling and pain that had resolved 2 days later.

Otherwise, he had no previous medical history, recent travels, tick bites, or illicit drug use. A 20-pack-year history of tobacco smoking and a daily alcohol consumption of 75–95 grams were reported. His vital signs were remarkable for heart rate of 110 beats/minute and oral temperature of 100.4 Fahrenheit. The physical exam showed decreased range of motion, erythema, swelling, and tenderness to palpation in both AC joints. Right third proximal interphalangeal (PIP) joint appeared swollen. The rest of the physical exam was benign.

Initial laboratory investigation included a white blood count of 14,800/mm^3^ (82% neutrophils, 4% lymphocytes, and 12% monocytes), erythrocyte sedimentation rate of 68 mm/h, and C-reactive protein of 34.6 mg/L (upper normal 0.30 mg/L). The remaining blood counts, biochemistry, urinalysis, HIV serology, and chest films were normal. After blood cultures were obtained, he was started empirically on ceftriaxone and vancomycin.

Bilateral shoulder plain radiographs did not reveal abnormalities. Ultrasound of the affected joints showed overlying anechoic fluid contiguous with the AC joints and periarticular soft tissue swelling with a thin rim of anechoic fluid in the right 3rd PIP. An arthrocentesis was performed, and grossly purulent fluid was drained from the right AC joint. Subsequent incision and drainage revealed gross purulence in both AC joints and in flexor sheath at the level of right third PIP. All these data were diagnostic for bilateral AC joint septic arthritis and right third PIP tenosynovitis.

Both AC synovial fluid and blood cultures grew* S. pneumoniae* susceptible to cefotaxime (minimum inhibitory concentration (MIC) 0.25**μ**g/mL), intermediately resistant to penicillin (MIC 0.064**μ**g/mL) and susceptible to vancomycin (MIC 0.38**μ**g/mL). Urine antigen was also positive for* S. pneumoniae*. The patient was continued on ceftriaxone, while vancomycin was stopped at day 3. A transthoracic echocardiography (TTE) failed to reveal any vegetation or valvular abnormalities.

He remained febrile for 1 week despite antibiotic therapy. Physical exam remained normal, including careful cardiovascular, neurologic, and fundoscopic evaluation.

**Figure 1:**
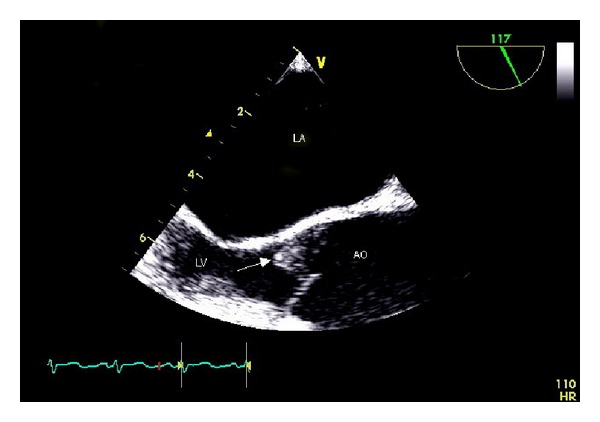
Two-dimensional transesophageal echocardiogram image of the aortic valve (arrow) showing an irregular 10-mm mass is attached to the left cusp of the aortic valve with an associated perforation. LA: left atrium; LV: left ventricle; AO: ascending aorta.

Repeated blood cultures on days 2, 3, 6, and 8 were all negative. Transesophageal echocardiogram (TEE) showed moderate aortic regurgitation, with an irregular and perforated 10-mm mass attached to the left cusp of the aortic valve (Figure 1).

Computed tomography of the head did not reveal septic emboli. A final diagnosis of IE with bilateral AC septic arthritis was made. Surgical aortic valve replacement was performed at day 12, as fever was persistent for more than 10 days despite antibiotic therapy. Native valve showed histopathology consistent with valve infection but did not reveal any organism.

The patient clinically improved after the surgery, with complete resolution of fever. Ceftriaxone was given for a total of 4 weeks, with no recurrence of his fever and slow recovery of his osteoarticular symptoms during 90 days of followup.

## 3. Discussion

Musculoskeletal manifestations are not uncommon in IE [2]. In a review of 9 studies of 1,312 patients with IE and musculoskeletal symptoms, 19–44% had at least one manifestation, mostly arthralgias and low back pain. Though, only 3.4% (*n* = 45) had documented osteoarticular infection (range: 0–15%) including septic arthritis and osteomyelitis [1]. Among these patients, the most common organisms reported were* Staphylococcus aureus* (23 cases), followed by* Streptococcus viridans* (8 cases) and enterococcus (4 cases). There was only one case that found* S. pneumoniae* as the causing organism. This was a 37-year-old woman, with history of IV drug use who was diagnosed with IE involving the mitral valve. Bone/gallium scan showed 2nd and 3rd costochondral joint involvement and blood cultures revealed* S. pneumoniae* [2]. Among the patients with IE and documented osteoarticular infection, multiple joints were usually affected, mostly the major joints of upper or lower extremity and the axial skeleton [1].

There was one case which mentioned IE with AC joint involvement, but no further clinical description was given [3].

Acromioclavicular joint septic arthritis has rarely been described. After an extensive review of the published literature, we found 30 documented cases of AC joint septic arthritis.  Table 1 describes the demographics, comorbidities, echocardiographic findings, causative organisms, and treatments administered in these cases. Out of 27 cases where an etiology was documented,* S. aureus* was the leading organism (52%).* S. pneumoniae* was isolated in 2 of these patients, both with hematologic malignancies [4, 5]. Echocardiography was reported in 4 cases and described IE in 2 of them [6–8]. One of these reports described a 74-year-old man who presented with unilateral AC joint septic arthritis caused by* S. aureus* and was ultimately found to have IE of the mitral and aortic valves, complicated by cerebellar septic emboli [6]. On the other report, a 41-year-old man initially presented with multiple joint involvement including bilateral AC joints. Cultures of the synovial fluid from his ankle as well as blood cultures were positive for a beta hemolytic group B* Streptococcus* and TTE indicated mitral valve IE [7].

*S. pneumoniae* accounts for less than 3% of etiology of IE [9]. Pneumococcal endocarditis is associated with poor outcomes due to the rapid destruction of endothelial tissue followed by valvular insufficiency, embolic complications, and eventually heart failure. Therefore, early diagnosis and treatment are crucial to prevent these complications [9, 10].

To our knowledge, this is the first documented case of bilateral AC joint septic arthritis as the manifestation of* S. pneumoniae* endocarditis. In summary, clinicians should recognize septic arthritis as a possible manifestation of IE, especially when an uncommon joint is involved or a rare organism is identified, as illustrated in this case.

## Figures and Tables

**Table 1:** Clinical and bacteriological features in previously reported cases of septic arthritis of the acromioclavicular joint.

Study	Sex	Age	Comorbidity/risk factors	Echocardiography result	Organism	Treatment
Good et al., 1978 [7] (bilateral)	M	41	None	TTE positive	GBS	Penicillin G + gentamicin

Adams and McDonald, 1984 [11]	—	57	Chronic steroid use, sarcoidosis	—	*Cryptococcus neoformans *	Surgical resection

Blankstein et al., 1985 [12]	M	48	Recent trauma	—	*S. viridans *	Antibiotic + surgical drain

Zimmermann et al., 1989 [13]	M	27	HIV	—	*S. aureus *	Surgical washout and resection of the distal clavicle + ciprofloxacin

Hughes et al., 1992 [14]	M	39	AIDS	—	Salmonella	Ciprofloxacin

Neault et al., 1996 [15]	M	26	Repair of a left type III AC joint separation with Dacron tape 5 years earlier	—	—	Surgical debridement and vancomycin

Widman et al., 2001 [4] (5 cases)	M	44	IV drug use, DM, hemodialysis	—	*S. aureus *	—
	M	41	Lymphoma treated but not on chemotherapy now	—	*S. pneumoniae *	—
	—	51	IV drug	—	*S. aureus *	—
	—	44	IV drug	—	*S. aureus *	—
	—	40	IV drug	—	*S. aureus *	—

Hammel and Kwon, 2005 [8]	M	68	DM	TEE negative	GBS	IV penicillin G

Laktasic-Zerjavic et al., 2005 [16]	M	44	DM	—	*S. aureus *	Antibiotic

Zicat et al., 2006 [17]	M	62	Knee replacement complicated by infection with the same pathogen	—	*S. aureus *	—

Chiang et al., 2007 [5]	F	55	MM, chemotherapy	—	*S. pneumonia *	Ceftriaxone + open AC joint resection and then linezolid
	F^a^	56	MM, chemotherapy	—	*S. viridans *	I & D and excision of distal clavicle + ceftriaxone
	F	79	—	—	GBS	Joint aspiration + ceftriaxone
	M	65	DM and renal insufficiency	—	—	Aspiration + pip/taz + nafcillin

Murdoch and McDonald, 2007 [18] (bilateral)	M	57	RA on prednisone and AZA, joint injection	—	MAI	I & D, azithromycin, moxifloxacin, ethambutol, and rifabutin

Tan et al., 2007 [19]	F	53	None, living in Singapore for 14 years	—	MTB	Rifampicin, isoniazid, ethambutol, and pyrazinamide

Battaglia, 2008 [20]	M	17	Trauma to shoulder followed by joint injection	—	*Ochrobactrumanthropi *	Irrigation and excision of distal clavicle + ciprofloxacin

Cone et al., 2008 [21]	M	63	DM	—	*S. aureus *	Surgical drainage + oxacillin

Iyengar et al., 2009 [22]	M	42	None	—	*S. aureus *	Flucloxacillin and oral fusidic acid

Bossert et al., 2010 [6] (5 cases)	M	74	DJD with preexisting cyst of AC joint	TEE positive	*S. aureus *	Oxacillin + gentamicin
	M	55	h/o dysmetabolic syndrome and gout	—	*S. aureus *	Oxacillin + ciprofloxacin
	M	64	COPD, RA not on DMARD	TTE negative	—	Oxacillin + ciprofloxacin
	M	38	IV drug use, hepatitis B and C, surgery several years earlier for fracture-dislocation	—	*S. aureus *	I & D, rifampin + ofloxacin
	M	62	AC joint steroid injection for pain	—	*S. aureus *	Ofloxacin and cloxacillin

Noh et al., 2010 [23]	M	63	DM	—	*S. aureus *	I & D and distal clavicle resection

Carey et al., 2010 [24]	M	65	None	—	*H. parainfluenzae *	I & D and levofloxacin

^a^Same patient listed above after 1 year.

AC: acromioclavicular; AZA: azathioprine; COPD: chronic obstructive pulmonary disease; DJD: degenerative joint disease; DM: diabetes mellitus; DMARD: disease-modifying antirheumatic drugs; GBS: group B *Streptococcus*; HIV: human immunodeficiency virus; I & D: incision and drainage; IV: intravenous; MAI: mycobacterium avium-intracellular; MM: multiple myeloma; MTB: mycobacterium tuberculosis; pip/taz: piperacillin/tazobactam; RA: rheumatoid arthritis; TEE: transesophageal echocardiography; TTE: transthoracic echocardiography; VAD: vincristine, doxorubicin, and dexamethasone.
